# The Medical Annual, 1917

**Published:** 1917-12

**Authors:** 


**The Medical Annual, 1917.** (Thirty-fifth year.) Pp. c. 662. Illustrated. Bristol: John Wright & Sons Ltd. 10s. net.

This hardy annual has appeared as usual, and apparently has not been affected by the war in so far as the subjects treated are concerned. It is as complete, carefully written and printed, and richly illustrated as heretofore, and no higher praise is possible.

